# *Pterygodermatites* (*Mesopectines*) *quentini* (Nematoda, Rictulariidae), a parasite of *Praomys rostratus* (Rodentia, Muridae) in Mali: scanning electron and light microscopy

**DOI:** 10.1051/parasite/2013032

**Published:** 2013-09-12

**Authors:** Malick Diouf, Yann Quilichini, Laurent Granjon, Cheikh Tidiane Bâ, Bernard Marchand

**Affiliations:** 1 Laboratory of Evolutionary Biology, Ecology and Management of Ecosystems, Faculty of Sciences and Techniques, Cheikh Anta Diop University of Dakar BP 5055 Dakar Senegal; 2 CNRS UMR 6134 SPE, Parasites and Mediterranean Ecosystems Laboratory, University of Corsica Pascal Paoli 20250 Corte France; 3 CBGP UMR IRD-INRA-CIRAD-Montpellier SupAgro, IRD Dakar Senegal

**Keywords:** Parasitic nematode, *Pterygodermatites*, *Mesopectines*, Rodent, Mali, SEM

## Abstract

*Pterygodermatites (Mesopectines) quentini* n. sp. (Nematoda, Rictulariidae) is described from the murine host *Praomys rostratus* in the south of the Republic of Mali. It differs from other species of the subgenus by the morphology of the head, which bears four simple cephalic papillae and a nearly axial oral opening, the number of caudal papillae, the number of precloacal cuticular formations, unequal spicules and the ratio of spicule lengths/body length. The use of scanning electron microscopy in combination with conventional light microscopy enabled us to give a detailed description of the morphological characters of this new species.

## Abstract (fr)

*Pterygodermatites (Mesopectines) quentini* n. sp. (Nematoda, Rictulariidae) est décrit du murinae *Praomys rostratus* dans le Sud de la République Malienne. Il diffère des autres espèces du sous-genre par la morphologie de sa tête, qui présente quatre papilles céphaliques simples et une ouverture orale pratiquement axiale, le nombre de papilles caudales, le nombre de formations cuticulaires précloacales et une inégalité des spicules et du ratio longueur des spicules/longueur du corps. L’utilisation de la microscopie électronique à balayage, ajoutée à celle de la microscopie optique conventionnelle, nous a permis de décrire avec précision les caractères morphologiques de cette nouvelle espèce.

## Introduction

Nematodes of the family Rictulariidae are divided into two genera, *Rictularia* Froelich, 1802 and *Pterygodermatites* Wedl, 1861 [9]. The buccal opening of the genus *Rictularia* is dorsally positioned and transverse with a single pharyngeal tooth, and the number of prevulvar armaments is lower than or equal to 34 pairs. In *Pterygodermatites*, the buccal opening is axial or slightly dorsal but never completely dorsal or transverse, with three pharyngeal teeth, and the number of prevulvar armaments ranges from 29 to 56 pairs. Based on different characters including the extent of the dorsal displacement of the buccal opening, the number of cephalic papillae and peribuccal denticles, arrangement of caudal papillae, and an increase in the number of prevulvar armaments, the species of *Pterygodermatites* are divided into five subgenera: *Paucipectines* Quentin, 1969, *Neopaucipectines* Quentin, 1969, *Pterygodermatites* Quentin, 1969, *Mesopectines* Quentin, 1969 and *Multipectines* Quentin, 1969 [8]. The subgenus *Mesopectines* has been observed in some Palearctic rodents with two evolutionary lines, one in the Ethiopian realm and one in the Oriental realm. The Asian species of this subgenus differ from the African species by an increase in the number of prevulvar and postvulvar cuticular armaments. To our knowledge two subgenera, *Neopaucipectines* and *Mesopectines*, have been recorded in African Muridae [1, 2, 5, 6, 11]. In the present study we describe a new species of *Pterygodermatites* (*Mesopectines*) and report its occurrence in the murine rodent *Praomys rostratus* Miller, 1900.

## Materials and methods

The nematodes studied herein were collected from *P. rostratus* captured in Piama in southeastern Mali, during a programme on the biodiversity in forest fragments of this area [7]. They were caught in February in riverine forest habitat. In total, 29 digestive tracts (21 males, 8 females) were examined under a stereo-microscope and helminth parasites were collected from the duodenum. They were fixed in 70% ethanol.

Nematodes were cleared in lactophenol and examined as wet mounts. Drawings were made with the aid of a drawing tube attached to a microscope. For scanning electron microscope studies, specimens were dehydrated in a graded ethanol series and dried using CO_2_ in an Emitech K850 critical point dryer. After being mounted, specimens were coated with gold/palladium in a Quorum Technologies SC7640 sputter coater and examined with a Hitachi S-3400N scanning electron microscope at acceleration voltages between 3 and 20 kV.

Four males and three females were examined for morphological studies. Measurements are given in micrometers unless otherwise indicated. The first measurement is that of the holotype (for male) or allotype (for female), followed by the range of the paratypes in parentheses. Type specimens have been deposited in the Muséum National d’Histoire Naturelle (MNHN), Paris, France.

## *Pterygodermatites (Mesopectines) quentini* n. sp.

urn:lsid:zoobank.org:act:20597A88-FB76-471C-AA49-4562CD5F98A0

Type-host: *Praomys rostratus* Miller, 1900 (Rodentia, Muridae) [3].

Type-locality: Piama 10° 87′ 08″N; 6° 10′ 16″W, in the southeast of the Republic of Mali.

Site of infection: duodenum

Type-material: Muséum National d’Histoire Naturelle, Paris, France, accession numbers: MNHN HEL321, holotype (male) and allotype (female); MNHN HEL322, paratypes. Collection date: February 2002.

Prevalence and intensity: 13.7% (29 examined, 4 infected); 4.2 (2–9) worms per host.

Etymology: dedicated to Jean-Claude Quentin.

### Description (Figures 1–3)

**Figure 1.**
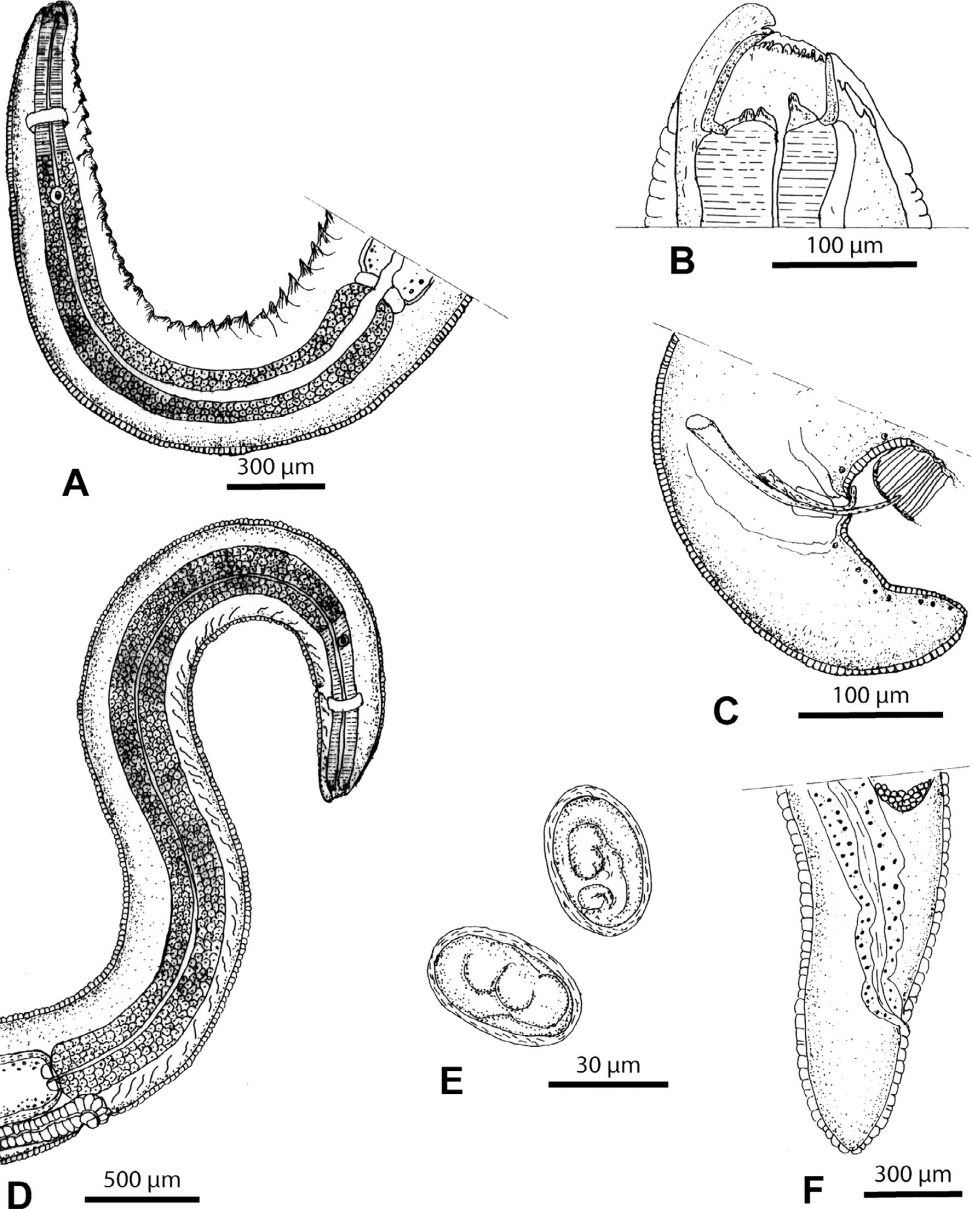
*Pterygodermatites* (*Mesopectines*) *quentini* n. sp. (A) Male, anterior extremity, right lateral view. (B) Male, cephalic extremity, right lateral view. (C) Male, posterior extremity, right lateral view. (D) Female, anterior extremity, right lateral view. (E) Embryonated eggs. (F) Female, posterior extremity.

**Figure 2.**
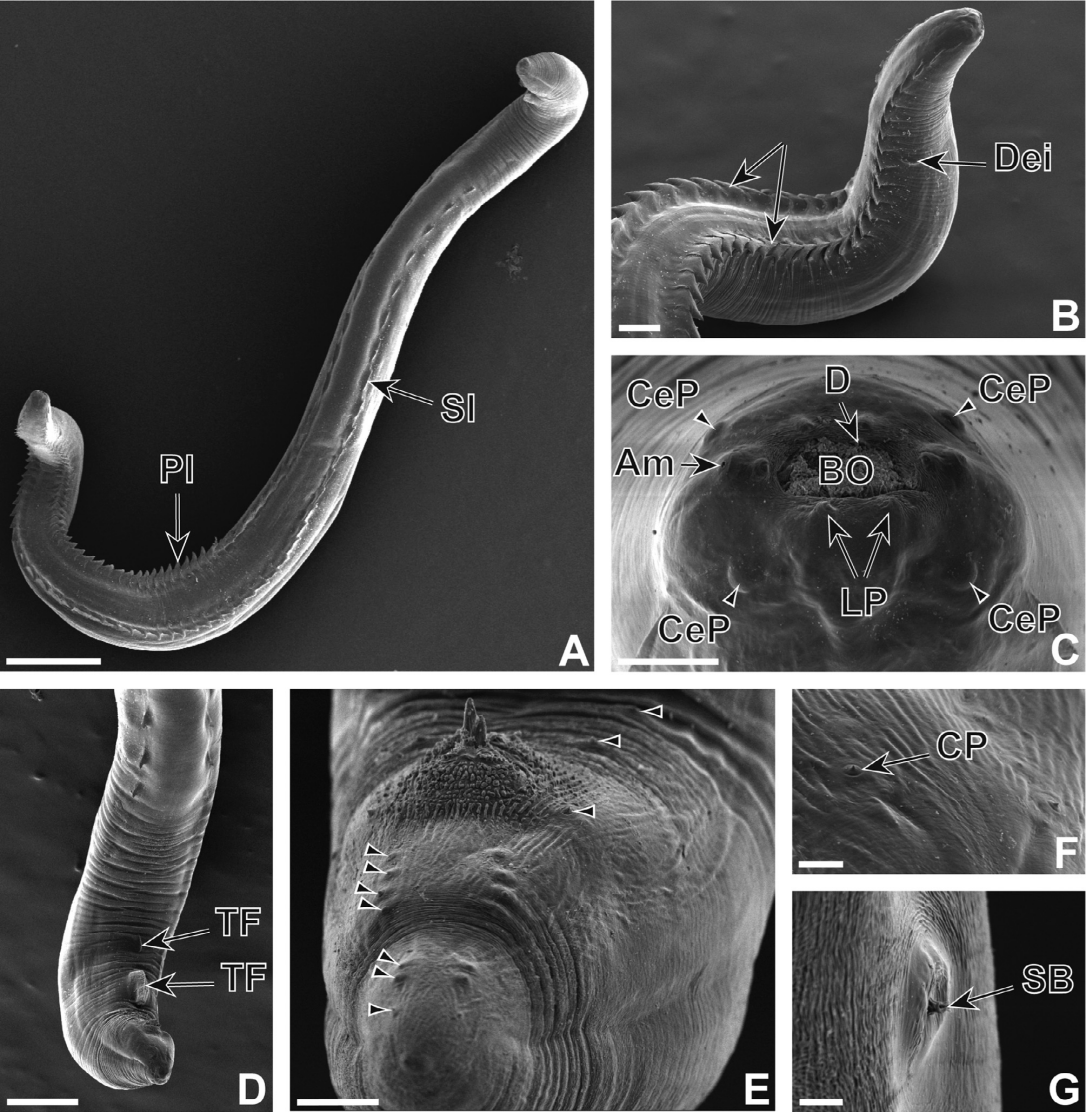
*Pterygodermatites* (*Mesopectines*) *quentini* n. sp. male. (A) Habitus, ventral view. (B) Anterior extremity, latero-ventral view. (C) Apical cephalic view. (D) Posterior extremity, latero-ventral view. (E) Posterior extremity, ventral view, note spicules protruding slightly from cloaca. (F) Caudal papillae. (G) Posterior extremity, sensorial bristle. Scales in μm: A, 500; B, 100; C, 30; D, 200; E, 30; F, 10; G, 5. Arrows indicate the two rows of plate-like structures. Arrowheads indicate caudal papillae. Am: amphid, BO: buccal opening, CeP: cephalic papillae, CP: caudal papillae, D: denticles, Dei: deirid, LP: labial papillae, Pl: plate-like structure, SB: sensory bristle, Sl: spine-like structure, TF tegumentary formation.

**Figure 3.**
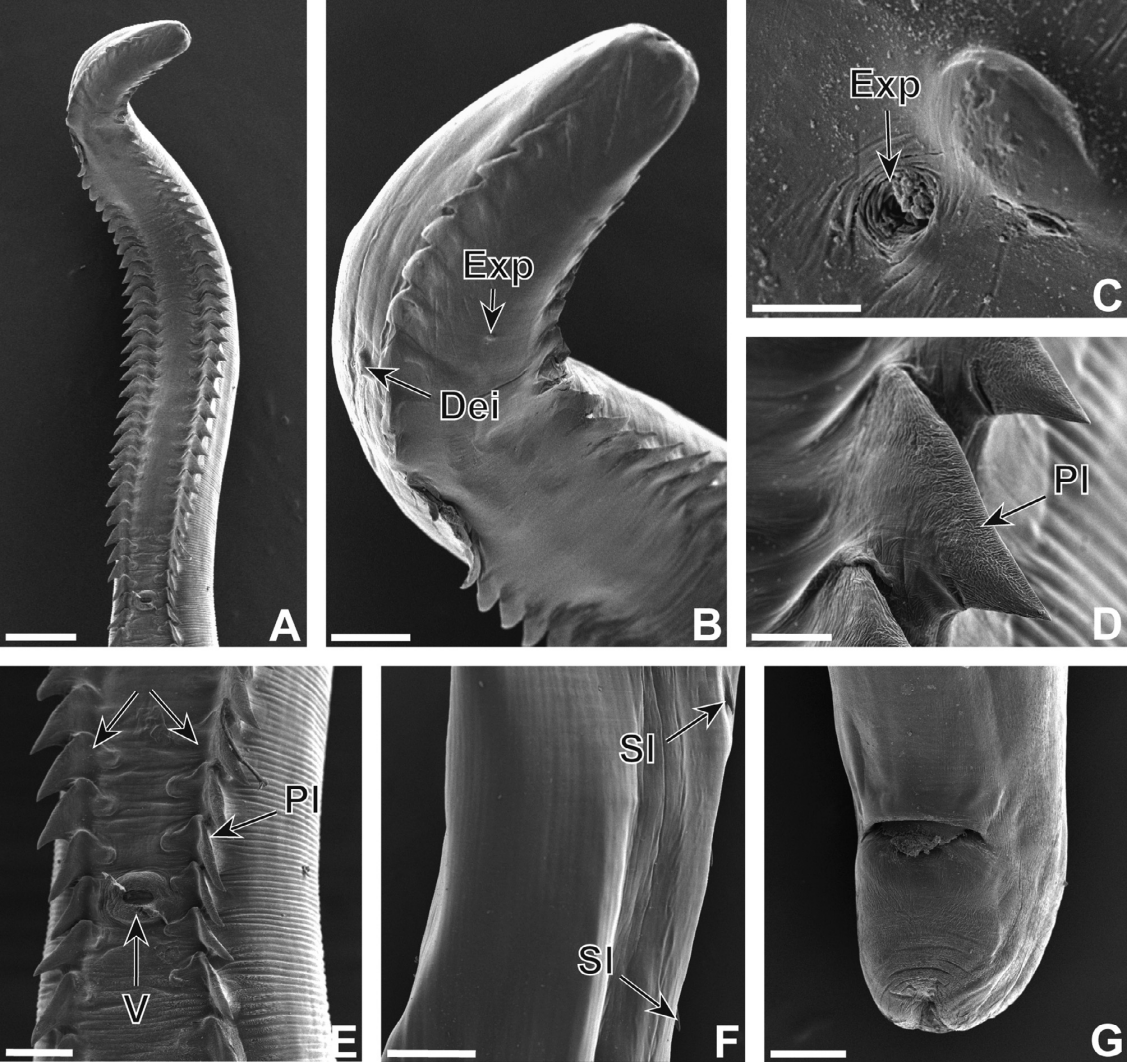
*Pterygodermatites* (*Mesopectines*) *quentini* n. sp., female. (A) Anterior extremity, ventral view. (B) Anterior extremity, latero-ventral view. (C) Excretory pore. (D) Plate-like cuticular armament. (E) Vulval opening, ventral view. (F) Posterior body part lateral view. (G) Tail, ventral view. Scales in μm: A, 300; B, 100; C, 5; D, 30; E, 100; F, 200; G, 100. Arrows indicate the two rows of plate-like structures. Dei: deirid, ExP: excretory pore, Pl: plate-like structure, Sl: spine-like structure, V: vulva.

Nematodes yellowish after fixation. Cephalic extremity with four simple cephalic papillae, two lateral amphids, six labial papillae in depressions (two ventral, two lateral at level of amphids and two dorsal) and circular oral opening with single crown of denticles (Figures 1B and 2C). Buccal capsule shifted slightly dorsally with one dorsal and two ventral pharyngeal teeth (Figure 1B). Cephalic protuberances present (Figures 1B and 2C). Two subventral rows of cuticular armaments along body, plate-like, juxtaposed in anterior part of body and spine-like, scattered in posterior part (Figures 1A, 2A, B and 3D–F). Oesophagus with short muscular part and long posterior glandular part (Figure 1A, D). Excretory pore, deirids posterior to nerve ring (Figure 1A, D). Transverse cuticular striations more or less regular, absent in posterior part of female (Figure 3F, G). Deirids with sensorial bristle.

#### Male

Length 5.4 (3.1–6.6) mm, width 250 (220–250). Number of buccal denticles 21(20–23). Nerve ring, excretory pore and deirids 250 (250–310), 300 (300–450) and 360 (360–500) from cephalic extremity, respectively (Figures 1A and 2B). Oesophagus 1.5 (1.4–1.9) mm long with muscular part 350 (350–475) long. In posterior extremity, variable number of precloacal cuticular formations (1–2) of different sizes (Figures 1C and 2D), on 280 (225–300) length of cuticle, in form of multiple associated cuticular folds anterior to first pair of caudal papillae (Figures 1C and 2D). Ten pairs of small caudal papillae (two precloacal, one adcloacal, seven postcloacal) (Figures 1C and 2E, F). Spicules unequal, left spicule 170 (152–170) long, right spicule 75 (72–85) long (Figures 1C and 2E). Gubernaculum 30 (15–30) long, width 20 (10–20) at base. In total 59 (58–61) pairs of cuticular armaments, including 13 (12–14) pairs of spine-like structures (Figure 2A). Last pair situated at 800 (750–850) from caudal extremity (Figure 2D). With scanning microscopy, one pair of sensorial structures was observed in the posterior part of the male at the level of the precloacal cuticular formations (Figure 2G).

#### Female

Length 25 (25–30) mm, width 500 (500–550). Number of buccal denticles 28 (28–30). Nerve ring, excretory pore (Figure 3B, C) and deirids 350 (330–360), 460 (450–560) and 600 (500–750) from cephalic extremity, respectively (Figures 1D and 3B). Oesophagus 3.6 mm (3.5–4) mm long with muscular part 600 (600–650) long; Vulva between two rows of cuticular armaments at 3.5 (2.5–3.8) mm from cephalic extremity (Figures 1D and 3A, E). Prevulvar armaments 41 (40–42) pairs and in total 70 (68–71) pairs of cuticular armaments. In anterior part plate-like structures, spaced 300 (300–750) posterior to vulvar opening (Figure 3D, E). Last pair situated at 900 (800–950) from the caudal extremity. Prominent vulvar opening. Uterus didelphic with branches posterior to vulva. Embryonated eggs ovoid 45 (43–45) long, 30 (28–30) wide (Figure 1E). Tail 350 (300–350) long (Figures 1F and 3G). Transverse cuticular striations absent in posterior third of body (Figure 3F).

## Discussion

Representatives of the subgenus *Mesopectines* parasitise rodents (Gerbillinae and Murinae), carnivores (Viverridae) and primates in both Africa and Asia [6, 8]. They are characterised by a buccal opening that is apical in position or somewhat dorsally displaced, three pharyngeal teeth, a single crown of regular peribuccal denticles, 37–51 pairs of prevulvar cuticular armaments and two subventral rows of caudal papillae [4, 10]. Our material conforms to this description. In the Ethiopian realm, five species have been described in this subgenus: *P.* (*M*.) *taterilli* Baylis, 1928, a parasite of *Taterillus gracilis* (Thomas, 1892) in Nigeria; *P.* (*M.*) *ortleppi* Quentin, 1969, a parasite of *Mastomys* sp. in Burkina Faso (formerly Haute Volta); *P.* (*M.*) *harrisi* Baylis, 1934, a parasite of *Mastomys coucha microdon* (Peters, 1852) in Tanzania (formerly Tanganyika) and *P.*(*M.*) *senegalensis* Diouf, Bâ & Marchand, 2000, a parasite of *Mastomys huberti* (Wroughton, 1909) in Senegal.

Among these species, only *P.* (*M.*) *taterilli*, *P.* (*M.*) *ortleppi* and *P.* (*M.*) *senegalensis* resemble our specimens with respect to the number of peribuccal papillae (6), the pharyngeal teeth and the number of prevulvar armaments (Table 1). *Pterygodermatites* (*M.*) *ortleppi* and *P.* (*M.*) *senegalensis* possess spicules that are equal. *Pterygodermatites* (*M.*) *taterilli*, the species closest to our specimens, also has spicules of unequal length. However, our material differs from *P.* (*M.*) *taterilli* by the number of cephalic papillae and caudal papillae, the ratio of spicule lengths/body length and the number of precloacal formations (Table 1). In *P.* (*M.*) *taterilli*, all precloacal formations (4) are well developed, contrary to our specimens. Similar to our specimens, *P.* (*M.*) *taterilli* has cephalic protuberances but its buccal opening is dorsal. *Pterygodermatites* (*M.*) *taterilli* has been reported numerous times in gerbilline rodents in several countries (Burkina Faso, Côte d’Ivoire, Senegal) [8]. Based on the differences outlined above, however, we conclude that our material belongs to a new species. Moreover, contrary to the remaining species in the subgenus *Mesopectines* which have been found in rodents inhabiting the savanna biome, the new species parasitises a forest rodent species.

**Table 1.** Morphometric characters of *Pterygodermatites* (*Mesopectines*) *quentini* n. sp. and the closest species.

Characteristics	*P.* (*M.*) *taterilli* Baylis, 1928	*P.* (*M.*) *ortleppi* Quentin, 1969	*P.* (*M.*) *senegalensis* Diouf, Bâ and Marchand, 2000	*P.* (*M.*) *quentini* n. sp. Present work
Male length (mm)	2.7	5.5	9.6 (8.9–11.5)	5.4 (3.1–6.6)
Female length (mm)	up to 40*	8.4	26 (24–28)	25 (25–30)
Number of cephalic papillae	8**	8	4	4
Number of precloacal cuticular formations	4	3	1	2
Spicule length (μm)	120/50*	67	100	170/75
Ratio of spicule length/body length	0.044/0.018	0.01	0.01	0.031/0.01
Pairs of caudal papillae	5***	10	8	10
Number of cuticular armaments in male	63	75	70 (69–72)	59 (58–61)
Number of prevulvar cuticular armaments in female	40–41	41	42 (40–43)	41 (40–42)

* According to Baylis (1928), the lengths of the female and the spicules are approximations.

** According to Quentin (1969).

*** According to Baylis (1928), this number might not necessarily reflect the total number of caudal papillae.
